# A rapid method for profiling of volatile and semi-volatile phytohormones using methyl chloroformate derivatisation and GC–MS

**DOI:** 10.1007/s11306-015-0837-0

**Published:** 2015-09-08

**Authors:** Catherine Rawlinson, Lars G. Kamphuis, Joel P. A. Gummer, Karam B. Singh, Robert D. Trengove

**Affiliations:** 1 Separation Science and Metabolomics Laboratory, Division of Research and Development, Murdoch University, Murdoch, WA 6150 Australia; 2 Metabolomics Australia, Murdoch University Node, Murdoch University, Murdoch, WA 6150 Australia; 3 CSIRO Agriculture Flagship, Private Bag No. 5, Wembley, WA 6913 Australia; 4 The UWA Institute of Agriculture, University of Western Australia, Crawley, WA 6009 Australia

**Keywords:** Jasmonic acid, Salicylic acid, Ethylene, Abscisic acid, Idole-3-acetic acid, Azelaic acid, Plant defence, *Medicago truncatula*

## Abstract

Phytohormones are central components of complex signalling networks in plants. The interplay between these metabolites, which include abscisic acid (ABA), auxin (IAA), ethylene, jasmonic acid (JA) and salicylic acid (SA), regulate plant growth and development and modulate responses to biotic and abiotic stress. Few methods of phytohormone profiling can adequately quantify a large range of plant hormones simultaneously and without the requirement for laborious or highly specialised extraction protocols. Here we describe the development and validation of a phytohormone profiling protocol, based on methyl-chloroformate derivatisation of the plant metabolites and analysis by gas chromatography/mass spectrometry (GC–MS). We describe the analysis of 11 metabolites, either plant phytohormones or intermediates of phytohormone metabolism; ABA, azelaic acid, IAA, JA and SA, and the phytohormone precursors 1-aminocyclopropane 1-carboxylic acid, benzoic acid, cinnamic acid, 13-epi-12-oxophytodienoic acid (13-epi-OPDA), linoleic acid and linolenic acid, and validate the isolation from foliar tissue of the model legume *Medicago truncatula*. The preparation is insensitive to the presence of water, facilitating measurement of the volatile metabolites. Quantitation was linear over four orders of magnitude, and the limits of detection between two and 10 ng/mL for all measured metabolites using a single quadrupole GC–MS.

**Electronic supplementary material:**

The online version of this article (doi:10.1007/s11306-015-0837-0) contains supplementary material, which is available to authorized users.

## Introduction

Phytohormones regulate important plant processes, including growth and development and response to biotic and abiotic stresses. They have been shown to act in complex signalling networks, where crosstalk signals a reaction to stress, such as attack by microbial pathogens and insect pests. Jasmonic acid (JA), salicylic acid (SA) and ethylene (ET) are thought to be central components of signalling pathways ultimately leading to the activation and fine tuning of defence responses; and abscisic acid (ABA) and auxin (indole-3-acetic acid; IAA) have also been implicated in defence against pathogens (Robert-Seilaniantz et al. 2011; Spaepen and Vanderleyden 2011). Both synergistic and antagonistic interactions between hormone signalling pathways have been reported to modulate defence to specific pathogens/pests (reviewed in Kunkel and Brooks 2002). To better understand the mechanisms of phytohormone activity, methods have been developed for the quantitation of these molecules and their regulators (reviewed in Schmelz et al. 2004).

Metabolomics analyses benefit from numerous analytical approaches to achieve maximum metabolome coverage. Volatile metabolites in particular can be lost during preparative stages within broader non-targeted metabolomics studies, if not specifically considered. Even within the complement of the plant metabolome termed phytohormones, the chemical diversity is such that complementary sample preparation and analytical methods be used for global phytohormone measurement (Boiero et al. 2007). The lower molecular weight and particularly higher volatility of ET for example, as compared to other commonly measured phytohormones renders this metabolite in particular, less amenable to global phytohormone quantitation. As a compromise to using an additional and specific method, solely for ET quantitation, Birkemeyer et al. (2003) and Schmelz et al. (2004) instead indirectly measured this phytohormone by way of quantitation of the amino acid precursor 1-aminocyclopropane 1-carboxylate (ACC). ACC has been demonstrated to have a direct correlation to ET production and as such is considered a marker of ET quantitation for global phytohormone measurement (Birkemeyer et al. 2003; Schmelz et al. 2004). ACC is the intermediate in the conversion of l-methionine to ET in the biochemical pathway of ET biosynthesis in plants (Caspi et al. 2014). This metabolite has since been analysed simultaneously with ABA, ABA-β-d-glucopyranosyl ester (ABA-GE), 24-epibrassinolide (BL), gibberellic acid A3 (GA3), IAA, JA and SA, thus allowing for more complete phytohormone coverage using a single gas chromatography/mass spectrometry (GC–MS) method (Birkemeyer et al. 2003). Müller and Munné-Bosch (2011) have used this same approach to measuring ET biosynthesis using liquid chromatography/mass spectrometry (LC–MS), again, to employ methods of phytohormone profiling incompatible with the quantitation of ET directly (Müller and Munné-Bosch 2011).

Chiwocha et al. (2003) also demonstrated the strength of LC/MS based phytohormone measurement by quantifying ABA, along with numerous IAAs, cytokinins and gibberellins, and their biochemical intermediates from lettuce (Chiwocha et al. 2003). More recently, Chen et al. (2012) developed a highly sensitive method to quantify phytohormones from rice leaves. Using a nano-LC approach, the assay benefited from a significant improvement in the lower limits of detection over higher flow LC systems, using as little as five milligrams of leaf tissue (Chen et al. 2012).

Common to both LC/MS and GC/MS metabolomics is the use of chemical modification or derivatisation of target metabolites, prior to instrumental analysis. Methods of phytohormone measurement by GC have particularly benefited from the conversion of these metabolites to their more volatile, thermally stable derivatives, with the most comprehensive GC–MS methods to date reporting the application of silylating agents to phytohormone profiling (Birkemeyer et al. 2003; Boiero et al. 2007; Engelberth et al. 2003; Muller et al. 2002; Schmelz et al. 2003, 2004).

As an alternative to silylation, Villas-Bôas et al. (2003), used the alkylating agent methyl chloroformate (MCF) to form stable derivatives of amino and non-amino organic acids, and validated its application to non-targeted metabolomics. Perrine et al. (2004) first applied this method of derivatisation to the measurement of IAA and tryptophan in rice, though the method was not expanded to include other phytohormones. MCF derivatisation, under alkaline conditions and in the presence of pyridine, was however found to be simple, high-throughput, and unlike silylation, derivatisation can proceed in the presence of water (Husek 1998; Smart et al. 2010; Villas-Bôas et al. 2003).

Although thorough, some of the more complete methods of phytohormone profiling have laborious and time-consuming extraction protocols, reducing throughput and potentially compromising optimal experimental design. So too, the lower limits of quantitation (LOQ) are often compromised in order to profile greater numbers of phytohormones. Thus there is a need for a method which can adequately quantify a large range of plant hormones simultaneously and within biologically relevant detection limits, whilst maintaining a reasonable degree of throughput in terms of instrumental and phytohormone extraction protocols.

A high throughput and sensitive method that would specifically identify a wide range of phytohormones and biochemical intermediates would benefit the plant science community. Toward this goal, we describe a simple GC–MS based approach to phytohormone quantitation, utilising a rapid and high-throughput extraction and derivatisation protocol for the measurement of various plant hormones and their precursors. The method uses MCF to derivatise metabolites and enhance non-polar extraction from plant tissues and was applied to phytohormone quantitation in the model plant *Medicago truncatula.*

*Medicago truncatula* has been used extensively to study plant–microbe interactions, not only for beneficial microbes like Rhizobia (Gough and Jacquet 2013) and mycorrhiza (Krajinski and Frenzel 2007), but also pathogens such as *Aphanomyces euteiches* (Hamon et al. 2010; Hilou et al. 2014), *Phoma medicaginis* (Kamphuis et al. 2008, 2012) and *Rhizoctonia solani* (Anderson et al. 2010; Anderson and Singh 2011), as well as insect pests (Kamphuis et al. 2013a; Kamphuis et al. 2013b). In addition, *M. truncatula* has been used to study certain aspects of plant development (Verdier et al. 2013) and responses to abiotic stress (Li et al. 2011; Wang et al. 2011), where plant hormones are key players and where the method described here will facilitate future studies. We show that the method can simultaneously quantify 11 metabolites, either plant phytohormones or their precursors [ABA, azelaic acid (AZ), IAA, JA and SA, and the phytohormone precursors ACC, benzoic acid (BA), cinnamic acid (CA), 13-epi-12-oxophytodienoic acid (13-epi-OPDA), linoleic acid and linolenic acid] from 100 mg amounts of plant tissue in *M. truncatula* with limits of detection between 2 and 10 ng mL^−1^ (7–30 mM).

## Materials and methods

### Chemicals

Reagents and standards including methyl chloroformate, pyridine, sodium bicarbonate, sodium hydroxide and sodium sulfate (anhydrous), n-alkanes (decane, dodecane, pentadecane, nonadecane, docosane, octacosane, dotriacontane, hexatriacontane), ABA, AZ, BA, CA, deuterated cinnamic acid (CA-d_6_), idole-3-acetic acid (IAA), JA, ACC, linolenic acid, linoleic acid and SA were purchased from Sigma-Aldrich (St Louis, MO, USA). 13-epi-12-oxophytodienoic acid (13-epi-OPDA) was purchased from Cayman Chemical (Ann Arbor, MI, USA). Chloroform (HPLC grade), and LC–MS grade methanol and water were purchased from Thermo Fischer Scientific (Scoresby, VIC, Australia).

### Plant preparation and growth

The *M. truncatula* accession Jester was used for development and proof of concept in this study. Jester is closely related to A17 with whom it shares 89 % of its genome. A17 is a derivative of the cultivar ‘Jemalong’ and the reference accession for the *M. truncatula* species. To ensure uniform germination, seeds were scarified using sandpaper and transferred to a Petri dish lined with blotting paper, and irrigated with sterile water. The seeds were kept at room temperature for 48 h; germinated seedlings were planted in Arabidopsis mix (Richgro Garden Products, Jandakot, Western Australia, 6164). Plants were grown in individual 0.9-L pots in growth cabinets with 16 h of light (22 °C) and 8 h of dark (20 °C) under high-pressure sodium and fluorescent lamps at 280 µE m^−2^ s^−1^ and tissue was harvested from 4-week-old plants.

### Phytohormone isolation and derivatisation

Leaves of *M. truncatula* were excised and immediately submerged in liquid nitrogen before storage at −80 °C. For the extraction of phytohormones, the leaf tissues were first ground to a fine powder in the presence of liquid nitrogen by mortar and pestle and 100 mg transferred to a two mL microcentrifuge tube. The extraction and combined derivatisation proceeded as described by Villas-Bôas et al. (2003), with some modification. To the ground tissue, 20 µL of 20 µg mL^−1^ deuterated cinnamic acid (CA-_d6_; in methanol) was directly added and the sample suspended in 200 μL of a sodium hydroxide (1 % w/v) solution. Added to the suspension were 147 µL of methanol and 34 µL of pyridine, before vigorous mixing by vortex for 25–30 s. Methyl chloroformate (20 µL) was then added and the suspension vigorously mixed for 25–30 s. A second volume of methyl chloroformate (20 μL) was added and the samples again mixed for 25–30 s. Subsequently, chloroform (400 µL) was added, the sample mixed for 10 s and a 50 mM sodium bicarbonate solution (400 µL) added. Following further mixing for 10–15 s, the extract was separated into two phases by centrifugation for 30 s at 16,100×*g*. The lower organic layer containing the phytohormones was transferred by pipette to a fresh microcentrifuge tube, being careful not to disturb the layer of plant debris separating the aqueous and organic layers. Anhydrous sodium sulphate was then added until the crystalline sodium sulphate appeared dry upon further addition. One hundred microlitre of the water-free solution was transferred to a glass analytical vial for GC–MS analysis.

### Calibration standards

Calibration standards were prepared, and subsequently derivatised by MCF as described, with minor modification. Individual standards were dissolved in methanol. A solution containing ABA, ACC, AZ, BA, CA, 13-epi-OPDA, IAA, JA, linolenic acid, linoleic acid and SA, each at 200 µg mL^−1^ was prepared and subsequently diluted in methanol for calibration stocks at the following concentrations: 0.02, 0.20, 2.00 and 20.00 µg mL^−1^. Three calibration levels were prepared from each stock solution (Supplementary Table 1).

The calibration solutions were prepared for analysis by addition to the 1 % NaOH solution already described. The metabolite standards and internal standard were added together with methanol to a total volume of 167 µL, and finally pyridine to maintain the 200:167:34 (1 % NaOH solution:methanol:pyridine) ratio described. MCF derivatisation proceeded as already described. Final calibration concentrations were 0.002, 0.005, 0.01, 0.02, 0.05, 0.1, 0.2, 0.5, 1, 2, 5, 10, 20 µg mL^−1^ after derivatisation and extraction (Supplementary Table 1).

### Gas chromatography/mass spectrometry

All method development was carried out on an Agilent 6890 gas chromatograph with a split/splitless injector and an Agilent 5973N quadrupole mass spectrometer with an EI source (Agilent, Palo Alto, CA, USA). The GC inlet was equipped with an Agilent focus liner with glass wool insert (SGE, Analytical Science Pty. Ltd.). A Varian Factor Four 5 ms column (30 m × 0.25 mm × 0.25 μm with a 10 m guard column) was used for all analyses (Varian, Palo Alto, CA, USA). Injection temperature and transfer-line were both 250 °C. A three µL injection was used with a 35 psi pressure pulse, held for 1 min. Chromatographic conditions had the column held at 40 °C for 1 min, and then temperature programmed at 20 °C min^−1^ to 320 °C, held for 2 min (17 min total). Helium was used as the carrier gas (1 mL min^−1^). The (electron ionisation; EI) ion source was maintained at 200 °C and solvent delay was 4.5 min. The MS was run in a scan mode (*m/z* 50–400) of acquisition for selection of appropriate EI mass fragments for each analyte. For quantitation methods, the MS was run in selected ion monitoring (SIM) mode with the respective ions presented in Table 1. The dwell time for each ion was 30 ms and each analyte had its own SIM window where possible. Methyl linoleate, methyl linolenate and methyl abscisate had the lowest cycle time of 1.83 cycles s^−1^. At basal biological levels, as determined during method development (using unchallenged *M. truncatula*), a minimum of 7–9 points across a peak was achieved and maintained for all analytes.

**Table 1:** The identifying features of the analytes resolved by these methods; accounting for the target phytohormones and related metabolites, isomers and multiple derivatisation products

Abbreviation	Name	CAS (underivatised analyte)	Retention indice	Retention time (min)	Quantifier ion (*m/z*)	Qualifier ion 1 (*m/z*)	Qualifier ion 2 (*m/z*)	SIM cycle (cycle/sec)
C10	Decane	124-18-5	1000	5.63	85	142	99	
MeBA	Benzoic acid, Me ester	65-85-0	1137	6.44	105	136	77	3.8
C12	Dodecane	112-40-3	1200	7.08	85	170	99	
MeSA	Salicylic acid, Me ester	69-72-7	1215	7.19	120	152	92	3.1
MeACC	1-aminocyclopropane-1-carboxylic acid, carbamate	22059-21-8	1267	7.55	141	109	82	3.1
MeCA-d6	d6-Cinnamic acid, Me ester	91453-04-2	1411	8.51	137	168	109	3.0
MeCA	Cinnamic acid, Me ester	140-10-3	1411	8.52	131	162	103	3.0
C15	Pentadecane	629-62-9	1500	9.13	99	212	113	
MeMeSA	Salicylic acid, Dimethyl ester	69-72-7	1523	9.3	135	165	92	2.7
MeAz	Azelaic acid, Me ester	123-99-9	1553	9.48	152	185	143	2.7
MeJA isomer 1	Jasmonic acid, Me ester isomer 1	77026-92-7	1655	10.02	151	224	83	3.8
MeJA isomer 2	Jasmonic acid, Me ester isomer 2	77026-92-7	1673	10.12	151	224	83	3.8
MeIAA	3-Indole acetic acid, Me ester	87-51-4	1863	11.32	189	103	77	3.8
C19	Nonadecane	629-92-5	1900	11.41	99	268	113	
Linoleic Acid, Me Ester	*cis,cis*-9,12-Octadecadienoic Acid, Me Ester	60-33-3	2106	12.40	67	81	94	1.8
Linolenic Acid, Me Ester	*cis,cis,cis*-9,12,15-Octadecatrienoic Acid, Me Ester	463-40-1	2114	12.43	79	95	108	1.8
MeABA isomer 1	Abscisic acid, Me Ester	14375-45-2	2129	12.51	190	162	134	1.8
C22	Docosane	629-97-0	2200	12.86	99	310	113	
MeABA isomer 2	Abscisic acid, Me Ester	14375-45-2	2205	12.89	190	162	134	3.8
13-epi-OPDA, Me Ester isomer 1	13-epi-12-oxophytodienoic acid, Me ester isomer 1	71606-07-0	2293	13.37	163	238	206	3.8
13-epi-OPDA, Me Ester isomer 2	13-epi-12-oxophytodienoic acid, Me ester isomer 2	71606-07-0	2306	13.45	163	238	206	3.8
C28	Octacosane	630-02-4	2800	16.21	85	99	71	

### Method validation

The described method was validated in three ways; extraction efficiency, limit of quantitation and intra and inter-day reproducibility.

The extraction efficiency was assessed by dividing a single ground sample of unchallenged *M. truncatula* foliar tissue among 12 replicate tubes; half of which were spiked with a phytohormone standard solution, for comparison against the remaining non-spiked controls. One hundred microlitre of a 20 ng µL^−1^ standard solution were spiked prior to the MCF derivatisation. The basal levels were determined for the non-spiked controls and the mean normalised intensity used as a baseline subtraction against the spiked. Baseline subtracted concentrations were determined against a calibration curve.

The LOQ was calculated from the derivatised standards and defined as the lowest concentration where a percent relative standard deviation (%RSD) of <10 (for *n* ≥ 6) was obtained, whilst maintaining a signal to noise (S/N) ratio >10. The limit of quantitation of each MCF derivative within *M. truncatula* sample matrix was determined on a sample by sample basis. This quantitation was defined by the concentration where the quantifier ion of each given MCF derivative measured a signal to noise ratio >10, whilst maintaining the ratio of qualifying ions to within 20 % of the values determined from the pure standard.

To test the reproducibility of all phytohormones, within and between extraction batches, ground *M. truncatula* foliar tissue was again divided among technical replicates and extractions performed over three consecutive days. Forty-eight individual MCF-preparations and analyses were prepared each day. These included 16 spiked with the phytohormone standards prior to MCF preparation, 16 non-spiked, 10 calibration standards and 8 standard dilutions. The spiked and non-spiked extractions were randomised among the GC–MS sequence with a 0.5 ng µL^−1^ calibration standard injected every fifth sample. This analytical sequence was repeated for three consecutive days, preparing fresh extractions daily. Before each sequence, a 10 cm length of the GC guard column was trimmed and the GC inlet liner replaced, consistent with internal routine preventative maintenance practices. The lowest level calibration sample was re-injected at the end of each analytical sequence, and again following preventative maintenance. Fifty injections were performed over each 24 h period.

### Data analysis

Data analyses were carried out using Agilent Chemstation software (v D.01.00). EI mass fragments for quantitation were selected based on their relative intensity and uniqueness (Table 1). Ion ratios between the main quantifier and qualifier ions were determined using pure standards. One quantifier ion and two qualifier ions were chosen for minimum reporting requirements.

To ensure accuracy at the lower LOQ, two calibration curves were constructed. The lower calibration was of concentrations downward from 5 µg mL^−1^; and the higher, upwards from 2 µg mL^−1^. Where an analyte was found to be close to the limit of detection (LOD), the higher calibration points were not used for quantitation.

## Results and discussion

Interplay between the biochemical pathways of phytohormone synthesis have long been established (Kunkel and Brooks 2002; Pilet 1965). Methods amenable to measuring all major classes of phytohormones however are essential to further understand their interplay, and robust, easy to use protocols of phytohormone quantitation are integral to ongoing efforts to characterise the role of these metabolites in plant stress responses and various aspects of plant development (Robert-Seilaniantz et al. 2011; Spaepen and Vanderleyden 2011).

Targeted metabolite profiling by GC–MS is efficient and reproducible, though current methods of phytohormone profiling by GC–MS can be complicated by use of specialised equipment (Schmelz et al. 2003, 2004), or are amenable to fewer classes of phytohormones (Table 2). To this end we developed and validated a method of metabolite extraction and derivatisation by MCF (Husek 1998; Smart et al. 2010; Villas-Bôas et al. 2003) for application to phytohormone quantitation. The sample preparation and derivatisation steps are presented schematically in Supplementary Fig. 1.

**Table 2:** Comparison of literature-cited methods of phytohormone profiling

Author, year	Metabolite^a^	Extraction	Sample matrix	Tissue mass	Instrument	Analytical run length	Extraction time	Notes
Pan et al. (2008)	Z, GA3, ICA, IAA, BA, ABA, MeIAA, SA, CA, MeBA, IBA, JA, MeSA, MeCA, MeJA, GA4, OPDA	SLE^a^	*Arabidopsis thaliana*, shoot	50-100 mg FW	LC–ESI–MS/MS	20 min	approximately 2.5 h per batch	Batch size undefined
Barkawi et al. (2010)	Auxins	SLE, 2 × SPE and derivatisation^b^	*Arabidopsis thaliana* seedling, tomato seedling root, maize inflorescence, *Saccharomyces cerivisiae*	25 mg FW	GC–EI–MS	17.5 min	3 h per 8 samples (manually)	Alternative automated protocol suggested with use of a robotic liquid handler
Schmelz et al. (2004)	SA, CA, JA, IAA, ABA, Linoleic acid, Linolenic acid,OPDA, COR	SLE, derivatisation and VPE^c^	*Arabidopsis thaliana*, shoot	100 mg FW	GC–CI–MS	25 min	6 h per 48 samples	Use of specialised equipment required for extraction of the phytohormones.
Muller et al. (2002)	SA, JA, IAA, ABA, OPDA	SLE, multiple SPE and derivatisation	*Arabidopsis thaliana*, shoot	20–200 mg FW	GC–CI–MS/MS	21 min	60 samples per day	
Chen et al. (2012)	Gibberellins, ABA, JA, IAA, IBA, SA	SLE, 2 × SPE, LLE and derivatisation^d^	*Oryza sativa*, leaf	5 mg FW	nano-LC–ESI–QToF–MS	74 min	ND	Highly specialised instrument for quantitation of phytohormones
Chiwocha et al. (2003)	Z, ZR, DPA, IAAsp, gibberellins, 2iP, ABA-GE, PA, IPA, 7′-OH-ABA IAA, ABA	SLE and SPE	*Lactuva sativa,* seed	50-100 mg DW	LC–ESI–MS/MS	30 min	approximately 26 h per batch	Batch size undefined, 24 h SLE step.
Perrine et al. (2004)	IAA, Trp	Simultaneous derivatisation and LLE	*Rhizobium,* exudate	1 mL supernatant	GC–EI–MS	14 min	approximately 15 min per sample	
Durgbanshi et al. (2005)	ABA, IAA, JA	SLE and LLE	*Citrus clementina*, mature leaf, *Hordeum vulgare*, seedlings	250–500 mg FW	LC–ESI–MS/MS	21 min	ND	
This study, 2015	BA, SA, ACC, CA, AZ, JA, IAA, Linoleic acid, Linolenic acid, ABA, 13-epi-12OPDA.	Simultaneous derivatisation and LLE	*Medicago truncatula*, shoot	100 mg FW	GC–EI–MS	17 min	20 samples per batch, 30–45 min per batch	

Only methods applied to the quantitation of two or more phytohormone-relevant metabolites were included

*Z* zeatin, *GA3/GA4* gibberellins, *ICA* indole-3-carboxylic acid, *BA* benzoic acid, *ABA* abscisic acid, *MeIAA* methyl indole-3-acetate, *SA* salicylic acid, *CA* cinnamic acid, *MeBA* methyl benzoate, *IBA* indole butyric acid, *JA* jasmonic acid, *MeSA* methyl salicylate, *MeCA* methyl cinnamate, *OPDA* oxophytodienoic acid, *COR* coranatine, *ZR* zeatin riboside, *ABA-GE* abscisic acid glucose ester, *7′-OH-ABA* 7′-hydroxy-abscisic acid, *PA* phaseic acid, *DPA* dihydorxyphaseic acid, *IAAsp* indole-3-aspartate, *2iP* isopentenyladenine, *IPA* isopentenyladenosine, *Trp* tryptophan, *ACC* 1-aminocyclopropane carboxylic acid, *IAA* indole-3-acetic acid, *AZ* azelaic acid, *13-epi-12-OPDA* 13-epi-12-oxophytodienoic acid, *FW* fresh weight, *DW* dry weight, *ESI* electrospray ionisation, *EI* electron ionisation, *CI* chemical ionisation

^a^Solid:liquid extraction; ^b^ solid-phase extraction; ^c^ vapour-phase extraction; ^d^ liquid:liquid extraction

Three key areas of method development were assessed; (1) the capacity of MCF to derivatise a number of phytohormones and phytohormone precursors, (2) the suitability of application to plant tissues, and quantitation of MCF-derivatives in a plant sample matrix (in our case foliar tissue from the model legume *M. truncatula*), and, (3) the cost, through-put and ease at which the method can be applied to routine phytohormone measurement. For comparison, this presented method was compared alongside current literature citations of phytohormone profiling (Table 2).

### Extraction and derivatisation

Ensuring the amenability of metabolites to routine analytical methods by way of chemical modification is common practice in targeted and non-targeted metabolite profiling. Silylation is the most common derivatising agent for GC–MS metabolomics, yet more volatile metabolites, for example BA, suffer from the requirement to dry the metabolite extract (data not shown).

We identified a combination of 11 metabolites, phytohormones, or their biochemical pathway intermediates; linolenoate, linoleote, 13-epi-12-oxo phytodienoate, ACC, indole-3-acetate, abscisate, azelate, benzoate, cinnamate, jasmonate and salicylate as amenable to MCF derivatisation and quantitation by GC–MS. The structures of the MCF derivatised metabolites are provided in Supplementary Fig. 2. Two additional phytohormones were of interest to this study, gibberellic acid (GA) and the cytokinin, zeatin (Z). GA, whilst amenable to the methods of isolation and derivatisation, is not well suited to GC analysis under the conditions most favourable to broader profiling (data not shown). Z on the other hand is not efficiently derivatised, likely due to steric hindrance of the functional groups required for MCF modification and as such is not amenable to the described methods. These two metabolites are well suited to measurement by LC–MS. Previous studies observed the efficiency of MCF derivatisation to be related to the alkalinity of the extract, with amino, di- and tricarboxylic acid yields being highest when the reaction mixture were above pH 10 (Villas-Bôas et al. 2003). Smart et al. (2010) recommended a pH higher than 12 for efficient MCF derivatisation. Additionally, Villas-Bôas et al. (2003) observed a general decrease in yield with longer reaction times in a non-targeted analysis of fungal metabolites.

**Fig. 1:**
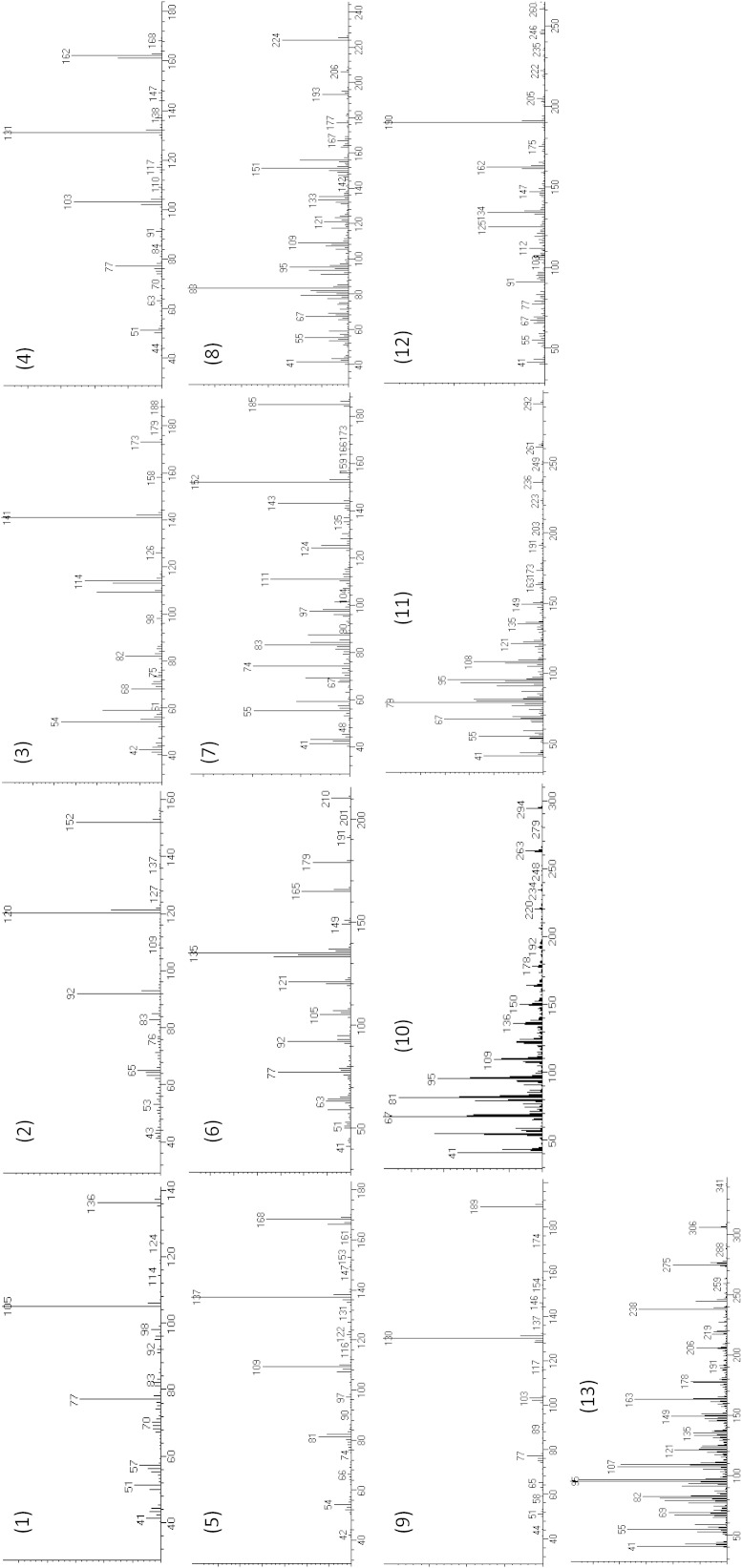
Mass spectrum for the methyl chloroformate derivatised products for (*1*) benzoic acid, (*2*) salicylic acid (methyl derivative), (*3*) 1-aminocyclopropane carboxylic acid, (*4*) cinnamic acid, (*5*) cinnamic acid-d6, (*6*) salicylic acid (dimethyl derivative), (*7*) azelaic acid, (*8*) jasmonic acid (isomer 1 and 2), (*9*) indole-3-acetic acid, (*10*) linoleic acid, (*11*) linolenic acid, (*12*) abscisic acid (isomer 1 and 2), (*13*) 13-epi-12-oxo phytodienoic acid (isomer 1 and 2). Isomers for jasmonic acid, abscisic acid and 13-epi-12-oxo phytodienoic acid resolve chromatographically but present the same mass spectrum in electron ionisation mode, a single mass spectrum is therefore presented

Our data demonstrated that pH and reaction time do not greatly influence the complete derivatisation of these 11 metabolites within the *M. truncatula* sample matrix, with no measured difference within one and four percent (w/v) NaOH solution, nor reaction times from 30 s to 5 min (data not shown). Of the phytohormones quantified, SA was the only analyte that formed multiple derivatisation products under the tested conditions. The predominant product, in all cases, resulted from the derivatisation of both the carboxylic acid and hydroxyl functional groups with the minor product only derivatising at the carboxylic acid functional group. The mass spectrum for all measured analytes is presented in Fig. 1.

Consistent with these preliminary analyses, and for confidence in achieving quantitative derivatisation of subsequent sample sets within this study, each were prepared with a mid-range calibration standard, and the metabolites extracted and derivatised in groups of no more than 20, allowing no sample to exceed 5 min reaction time.

### Quantitation

Technical variability can be introduced into the analysis during extraction, derivatisation or instrumental analysis. Sample matrix may also interfere with the yield of derivatised products. In this method, we use isotopically labelled cinnamic acid (CA-_d6_) to compensate for these possible inconsistencies. Normalisation of individual analyte responses to this internal standard can partially correct unavoidable variability. CA-_d6_ was spiked directly into the ground plant foliar tissue, before extraction, to best mimic the recovery and derivatisation of the analysed compounds. The deuterated CA internal standard provided reproducibility with a single MCF-derivatisation product, and mass spectrum easily distinguished from the naturally occurring analogue.

**Fig. 2:**
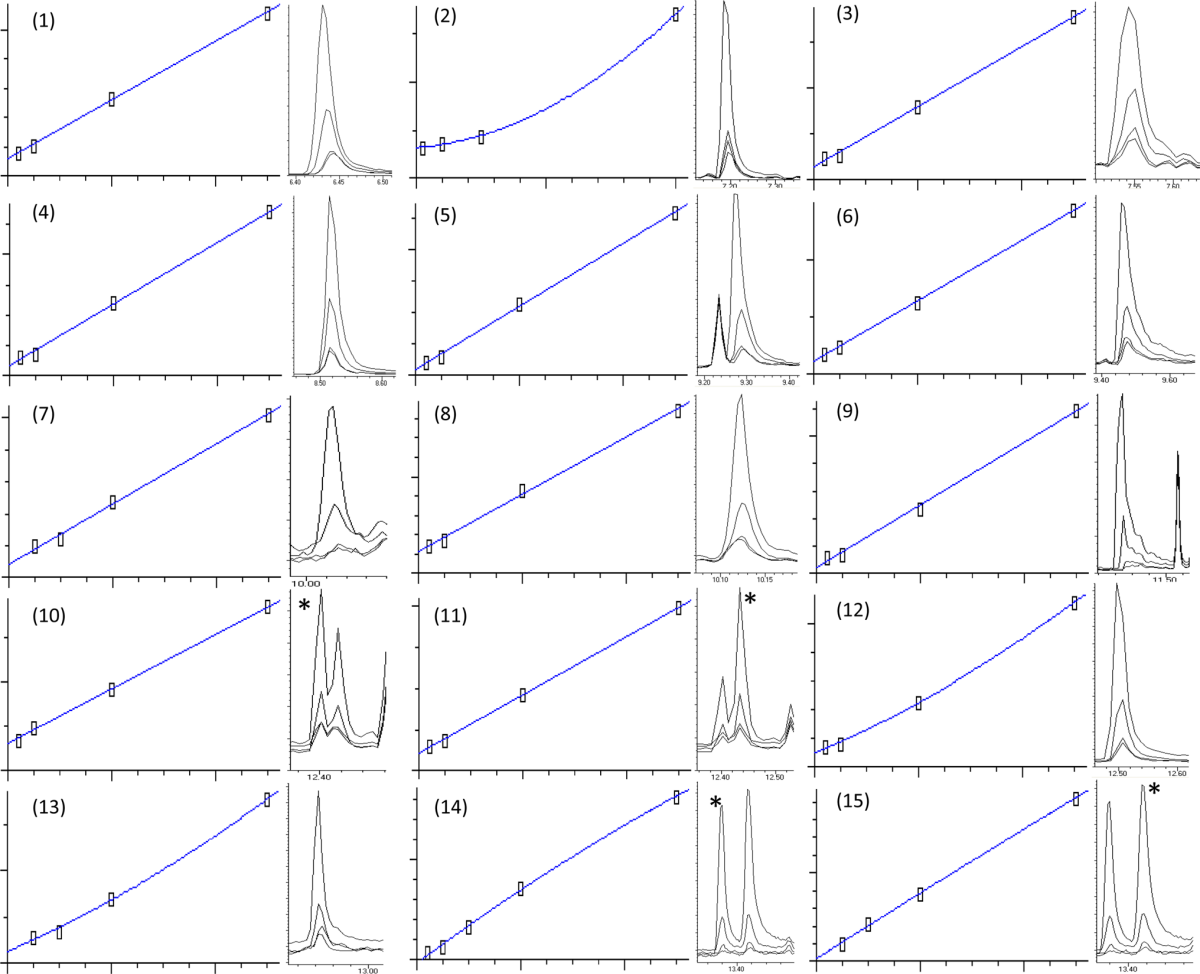
Four lowest calibration standards and the chromatogram overlay for: (*1*) MeBA—R^2^ 0.999, 0.002–0.05 µg mL^−1^, (*2*) MeSA—R^2^ 0.999, 0.005–0.2 µg mL^−1^, (*3*) MeACC—R^2^ 0.999, 0.002–0.05 µg mL^−1^, (*4*) MeCA—R^2^ 0.998, 0.002–0.05 µg mL^−1^, (*5*) MeMeSA—R^2^ 0.999, 0.002–0.05 µg mL^−1^, (*6*) MeAz—R^2^ 0.999, 0.002–0.05 µg mL^−1^, (*7*) MeJA isomer 1—R^2^ 0.997, 0.005–0.05 µg mL^−1^, (*8*) MeJA isomer 2—R^2^ 0.997, 0.002–0.05 µg mL^−1^, (*9*) MeIAA—R^2^ 0.999, 0.002–0.05 µg mL^−1^, (*10*) linoleic acid, Me Ester—R^2^ 0.999, 0.002–0.05 µg mL^−1^, (*11*) linolenic acid, Me Ester—R^2^ 0.999, 0.002–0.05 µg mL^−1^, (*12*) MeABA isomer 1—R^2^ 0.999, 0.002–0.05 µg mL^−1^, (*13*) MeABA isomer 2—R^2^ 0.999, 0.005–0.05 µg mL^−1^, (*14*) Me-13-epi-OPDA isomer 1—R^2^ 0.999, 0.002–0.05 µg mL^−1^, (*15*) Me-13-epi-OPDA isomer 2—R^2^ 0.999, 0.005–0.05 µg mL^−1^. *Asterisked peaks* represent the analyte of interest for that calibration

Most analytes were linear over four orders of magnitude or greater (data not shown). The highest calibration standard for MeBA and MeIAA overloaded the MS detector, so quantitation was limited to a maximum concentration of 10 µg mL^−1^. For accurate quantitation of less abundant phytohormones the calibrations at the lower LODs are of particular importance. Eight of the 11 metabolites had a measured linearity within two and 50 ng mL^−1^ (r^2^ ≥ 0.997; Fig. 2).

All metabolites, except for 13-epi-OPDA were reproducibly detected at 0.005 ng/µL or lower (Table 3). All of the metabolites had a calculated  %RSD less than 10 (*n* ≥ 6). This reproducibility would be improved with the availability of purer metabolite standards. As an example, ABA, JA and 13-epi-OPDA were purchased as a *cis*- and *trans*- mix of isomers which chromatographically resolve by these methods (Table 3). However, it is the sum of these isomers which represent the prepared concentration. Isomers of the same metabolite were therefore quantified using the same ions to allow calibration and sample responses to be summed for absolute quantitation.

**Table 3:** The determined limits of quantitation (LOQ, ng µL^−1^ and mM), extraction efficiencies, and the intra- and inter-day reproducibility of the MCF-derivatized phytohormones/phytohormone precursor (in order of elution)

Compound	LOQ ng μL^−1^ (mM)	RSD at LOQ (%)	Recovery (%)	Recovery RSD (%)	Intra-day reproducibility RSD (%)	Inter-day reproducibility RSD (%)
MeBA	0.002 (14.7)	8.4	96.0	6.6	4.9	18.9
MeACC	0.002 (11.6)	3.6	79.3	3.8	10.3	16.6
MeCA	0.002 (12.3)	9.6	97.7	4.4	4.9	14.2
MeCA_-d6_ (IS)	–	–	99.0	1.6	–	–
MeMeSA	0.005 (30.1)	9.1	67.4	5.8	12.3	22.3
Methyl Azelate	0.005 (23.1)	9.1	84.5	5.2	10.9	17.4
MeJA Isomer 1	0.02 (89.2)	7.0	88.0	9.4	28.4	21.3
MeJA Isomer 2	0.002 (8.9)	9.8	91.9	3.3	13.3	16.3
MeIAA	0.002 (10.6)	8.8	99.1	4.0	7.3	18.9
Linoleic Acid, Me Ester	0.002 (6.8)	6.8	55.7	4.9	8.9	17.4
Linolenic Acid, Me Ester	0.002 (6.8)	7.7	86.1	10.5	8.7	15.5
MeABA Isomer 1	0.005 (18.0)	8.6	102.1	3.9	6.6	17.9
MeABA Isomer 2	0.02 (71.9)	3.2	109.8	3.6	9.5	24.6
13-epi-OPDA, Me Ester Isomer 1	0.01 (32.6)	9.8	55.6	15.7	10.7	15.3
13-epi-OPDA, Me Ester Isomer 2	0.01 (32.6)	4.1	59.4	14.8	14.4	18.2

The intra-/inter-day reproducibility was determined within the *M. truncatula* metabolite matrix

LOQ was determined by an RSD (%) ≤10 (*n* = 6). The higher LOQ of the minor derivatisation product of SA was due to only representing a very small part of the total SA and has therefore been omitted

Instrument capabilities are reflected in the detection limits achieved by analysis of pure standards, whereas plant matrices present a complex and dynamic system for GC–MS analysis. Consequently, the detection limit of pure standards will typically be lower than can be achieved within plant matrix. Muller et al. (2002) synthesised five isotopically labelled phytohormones (SA, JA, IAA, ABA and OPDA) and determined their respective detection limits as standards, and compared these values to when the labelled phytohormones were spiked into an extract from *Arabidopsis thaliana*. The lowest concentration that meets detection limit criteria of minimum S/N for these standards within plant matrix was 11.5–15 times greater when compared to pure standards (Muller et al. 2002).

### Recovery

As several of the phytohormones are known to be present at basal levels within plant tissues, recovery was calculated by the analysis of six replicates of unchallenged *M. truncatula* foliar tissue extract, against a further six replicates spiked with 100 µL of a 5 ng µL^−1^ solution of the metabolite standards. A recovery of 75 % or better was achieved for nine of the 11 metabolites (Table 3). This route of analysis showed a high degree of reproducibility as all analytes (except linolenic acid, methyl ester and 13-epi-OPDA, methyl ester) showed  %RSD’s below 10. The high  %RSD of linolenic acid was likely an artefact of the high endogenous level of this compound within *M. truncatula* tissue, and therefore working closer to the upper limit of quantitation of this metabolite.

### Intra- and inter-day reproducibility

Metabolomics analyses produce multi-dimensional datasets, combining numerous treatment groups, biological replication, quality controls, and often longitudinal data. Batch variation adds further dimension to particularly large studies, introduced through extraction batches and preventative instrument maintenance. Previous work demonstrated the excellent stability of MCF derivatives of 26 amino and non-amino organic acids when stored at room temperature for 72 h (RSD <10 %) (Villas-Boas et al. 2011). This method however presents a high volume injection of a plant matrix into the GC inlet, potentially making it particularly susceptible to intra- and inter-day variation. In this work, we therefore sought to examine the intra and inter-day reproducibility of the MCF preparation and GC–MS analysis of the 11 phytohormones. The intraday reproducibility was calculated from the 16 spiked foliar preparations, analysed among a sample sequence of 50 injections, with the reported RSDs capturing the entire extraction and derivatisation, through to analytical variation. The inter-day measurements represent the reproducibility of the entire method repeated over three consecutive days, whilst also capturing the variability introduced through daily preventative instrument maintenance (Table 3). These presented methods achieved a RSD of less than 15 % for ten of the 11 phytohormones, with only the minor isomer of JA exceeding this value for intraday reproducibility. The inter-day reproducibility was below 20 % RSD for eight of the 11 phytohormones. Though still within an acceptable RSD, the remaining three phytohormones, SA, JA and ABA, which reported a higher RSD, likely suffered in reproducibility due to representation by multiple isomers or derivatisation products, and the consequential division of intensity amongst multiple peaks. The added robustness of the intraday analyses over the inter-day also demonstrate the particular reliability of this method to phytohormone preparation and derivatisation, more specifically.

## Concluding remarks

The complex interplay between various phytohormones is crucial for many plant processes including response to biotic and abiotic stress. Therefore it is important to be able to easily and accurately measure changes in these compounds under various conditions where sample material may be limited. Here we have presented a very quick and robust method for the analysis of 11 metabolites; phytohormones or their biochemical pathway intermediates by GC–MS, and demonstrated application using the foliar tissue of *M. truncatula*, requiring only 100 mg of plant tissue. The method presents a high degree of reproducibility in the isolation of these target metabolites, and calibration linearity in the range of four orders of magnitude. This protocol is particularly high through-put in terms of sample preparation and instrumentation, requires no specialised equipment for phytohormone isolation and preparation and therefore samples can be generated in a wide range of laboratories and enables analysis of large, highly replicated experiments in a short turnaround time.

Metabolites with higher volatility including many phytohormones, require particular consideration during preparative processes, but are an important component of the plant metabolome. The application of targeted methods such as these towards achieving wider metabolome coverage will benefit non-targeted metabolomics data sets. The plant science and metabolomics community would also be well served by the application of these methods to other species and tissue types and even the extension to non-targeted data acquisition methods, such as those validated by Villas-Bôas et al. (2003) and Smart et al. (2010) for a more comprehensive picture of the plant metabolome.

## Electronic supplementary material

Below is the link to the electronic supplementary material.

Supplementary material 1 (DOCX 24 kb). Supplementary Fig. 1. The preparative workflow of the described methods of pytohormone isolation and derivatisation

Supplementary material 2 (DOCX 66 kb). Supplementary Fig. 2 Chemical structures of the 11 phytohormone MCF-derivatives

Supplementary material 3 (DOCX 18 kb). Supplementary Table 1 Preparation of the calibration standard curves. All preparations maintained the described 200:167:34 (1 % NaOH:methanol:pyridine) ratio
