# Supplementary material for: A rapid method for profiling of volatile and semi-volatile phytohormones using methyl chloroformate derivatisation and GC–MS
Source: Metabolomics. 2015 Sep 8;11(6):1922–33. doi: 10.1007/s11306-015-0837-0 (PMC4605965; doi:10.1007/s11306-015-0837-0)
Supplement: Supplementary file 1 — Supplementary material 1 (DOCX 24 kb). Supplementary Fig. 1. The preparative workflow of the described methods of pytohormone isolation and derivatisation [file 11306_2015_837_MOESM1_ESM.docx]

Step 1

Step 5

Step 2

Step 4

Step 3

Homogenise plant material under liquid nitrogen.

Spike d6-Cinnamic acid directly on to homogenate.

Suspend in 1% NaOH:MeOH:pyridine (200:167:34)

Add chloroform and sodium bicarbonate solution.

Centrifuge sample and aliquot lower organic layer for analysis.

Step 6

Dry with anhydrous sodium sulfate.

Supplementary Fig 1. The preparative workflow of the described methods of phytohormone isolation and derivatisation.
