# Supplementary material for: A rapid method for profiling of volatile and semi-volatile phytohormones using methyl chloroformate derivatisation and GC–MS
Source: Metabolomics. 2015 Sep 8;11(6):1922–33. doi: 10.1007/s11306-015-0837-0 (PMC4605965; doi:10.1007/s11306-015-0837-0)
Supplement: Supplementary file 2 — Supplementary material 2 (DOCX 66 kb). Supplementary Fig. 2 Chemical structures of the 11 phytohormone MCF-derivatives [file 11306_2015_837_MOESM2_ESM.docx]

13-epi-12-oxo phytodienoate, methyl ester

(13-epi-OPDA, Me Ester)

Methyl Abscisate (MeABA)

Methyl Salicylate, methyl ether (MeMeSA)

Methyl Linolenoate

Methyl Linoleote

Methyl Indole-3-acetate (MeIAA)

Methyl Jasmonate (MeJA)

Methyl Azelate (MeAZ)

Methyl Salicylate (MeSA)

Methyl Cinnamate (MeCA)

1-aminocyclopropane carboxylate, carbamate (MeACC)

Methyl Benzoate (MeBA)

Supplementary Fig 2. Chemical structures of the 11 phytohormones MCF derivative
