# Supplementary material for: A rapid method for profiling of volatile and semi-volatile phytohormones using methyl chloroformate derivatisation and GC–MS
Source: Metabolomics. 2015 Sep 8;11(6):1922–33. doi: 10.1007/s11306-015-0837-0 (PMC4605965; doi:10.1007/s11306-015-0837-0)
Supplement: Supplementary file 3 — Supplementary material 3 (DOCX 18 kb). Supplementary Table 1 Preparation of the calibration standard curves. All preparations maintained the described 200:167:34 (1 % NaOH:methanol:pyridine) ratio [file 11306_2015_837_MOESM3_ESM.docx]

Supplementary Table 1. Preparation of the calibration standard curves. All preparations maintained the described 200:167:34 (1% NaOH:methanol:pyridine) ratio.

| Calibration standard (ng/µL) | 0.02ng/µL (µL) | 0.2ng/µL (µL) | 2ng/µL (µL) | 20ng/µL (µL) | 200ng/µL (µL) | Methanol (µL) | 1% NaOH (µL) | Pyridine (µL) |
| --- | --- | --- | --- | --- | --- | --- | --- | --- |
|  |  |  |  |  |  |  |  |  |
| 0.002 | 40 |  |  |  |  | 107 | 200 | 34 |
| 0.005 | 100 |  |  |  |  | 47 | 200 | 34 |
| 0.01 |  | 20 |  |  |  | 127 | 200 | 34 |
| 0.02 |  | 40 |  |  |  | 107 | 200 | 34 |
| 0.05 |  | 100 |  |  |  | 47 | 200 | 34 |
| 0.1 |  |  | 20 |  |  | 127 | 200 | 34 |
| 0.2 |  |  | 40 |  |  | 107 | 200 | 34 |
| 0.5 |  |  | 100 |  |  | 47 | 200 | 34 |
| 1 |  |  |  | 20 |  | 127 | 200 | 34 |
| 2 |  |  |  | 40 |  | 107 | 200 | 34 |
| 5 |  |  |  | 100 |  | 47 | 200 | 34 |
| 10 |  |  |  |  | 20 | 127 | 200 | 34 |
| 20 |  |  |  |  | 40 | 107 | 200 | 34 |

**A rapid method for profiling of volatile and semi-volatile phytohormones using methyl chloroformate derivatisation and GC-MS**

**Metabolomics**

Catherine Rawlinson, Lars G. Kamphuis, Joel P. A. Gummer, Karam B. Singh and Robert D. Trengove^1^

1 Author for correspondence. Email: R.Trengove@murdoch.edu.au. Fax: +6189360 6686. Phone: +6189360 7639
